# Halved but Potent: Exploring the Inhibitory Property of Curcumin Derivatives Against Evolving SARS-CoV-2 Strains

**DOI:** 10.1007/s10930-025-10309-1

**Published:** 2025-12-16

**Authors:** Atala Bihari Jena, Umesh Chandra Dash, Asim K Duttaroy

**Affiliations:** 1 National Centre for Cell Science, Savitribai Phule Pune University Campus, Ganeshkhind, Pune India; 2 School of Biotechnology, Kalinga Institute of Industrial Technology (KIIT) Deemed to be University, Campus 11, Bhubaneswar, Odisha 751024 India; 3 Department of Nutrition, Faculty of Medicine, Institute of Medical Sciences, University of Oslo, Oslo, Norway

**Keywords:** SARS CoV-2, B.1.1.7, D614G, In silico, Half-curcumin

## Abstract

In the prevailing pandemic, the emergence of several variants has created concerns, as none of the vaccines have 100% effectiveness. As a countermeasure to combat the infection of SARS-CoV-2 and its variants and to minimize the morbidity, alternative therapeutics have been prioritized, mainly including phytocompounds. In our recent studies, we have considered polyphenols, such as catechin, curcumin, and 8-Hydroxydihydrosanguinarine (8-HDS), for this purpose and found their tendency to bind to the receptor-binding domain of S-protein, thereby inhibiting viral infiltration into the cell. In the current scenario, with the appearance of multiple viral strains, we have furthered our studies on two highly infectious variants of SARS-CoV-2: D614G and B.1.1.7, commonly identified as the UK variant. Our research incorporates various computational methodologies to decipher the potential role of our previously studied phytocompounds and the curcumin derivative, half-curcumin, inhibiting viral attachment and entry for both D614G and the B.1.1.7 lineage. The physicochemical characterization of the spike (S) protein of the UK variant suggests it is less thermostable, which raises questions about its transmissibility in warmer regions of the globe. All the studied phytocompounds have an immense ability to hinder the S protein interaction towards the human cell receptor ACE2 for both the variants. The half-curcumin has also potential drug suitability, which is confirmed through its characterization of ADME (Physicochemical, Water Solubility, Lipophilicity, Drug-likeness, and Pharmacokinetics) properties, and can be considered as a future therapeutic molecule for anti-COVID-19 drug design.

**Graphical Abstract:**

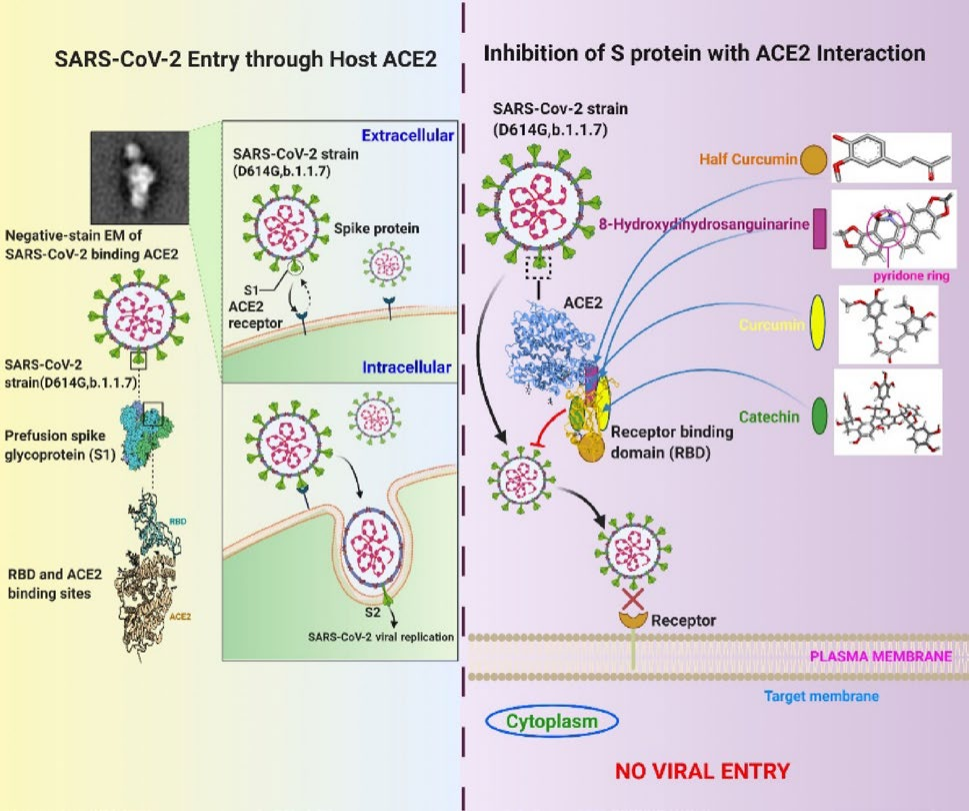

## Introduction

The pervasiveness of epidemic and pandemic viruses is often associated with multiple mutations that result in structural changes and enhanced transmissibility, occasionally leading to increased severity. Evolutionary mutations in other coronaviruses, alpha viruses, and filoviruses have had a significant impact in recent history. Their mutations resulted in greater pathogenicity and antigenicity, as well as the ability to infect additional hosts. Though most viral mutations are short-lived and result from deletions, the persistent ones are propitious to viral fitness and potentiate viral proliferation. It had to deal with vaccine development and medicine formulation as well. [1]

RNA viruses are more mutable than the host cells and other DNA viruses [1, 2], which has great impact on their life cycle, adaptability and transmissibility to the host. Consequently, emergence of several mutants and variants were discerned in several important (infectious) RNA viruses like, Chikungunya virus [3], ebola virus [4], avian influenza H_5_N_1_ [5], SARS-CoV [6] and MERS-CoV [7]. SARS-CoV-2, responsible for the recent COVID-19 outbreak, is a highly adaptable positive-sense single-stranded RNA virus belonging to the Coronavirinae subfamily, Coronaviridae family, and Nidovirales order, and is classified as a Betacoronavirus [8]. As of 23rd August 2021, more than 90 million infected cases were confirmed across the globe with Surpassing 4 million lives lost (www.covid19.who.int).

The ~ 30 kb long genome has several small open reading frames (ORFs), located in the 3’ region, encode for the replication of structural poly-proteins, i.e. envelope (E), nucleocapsid (N), spike (S), membrane (M), and other non-structural proteins (NSP) of the virus cell [9–12]. The S protein of SARS-CoV-2 functions as a glycoprotein that contains 1273 amino acids, forming a trimeric spike on the virion surface and is composed of S1 and S2 subunits [13]. The S1 subunit attaches to the receptor, while the S2 subunit facilitates membrane fusion [14]. The viral spike protein, a glycoprotein, interacts with the human receptor ACE2 (angiotensin-converting enzyme 2). Compared to the SARS-CoV spike protein, the S protein’s receptor-binding domain (RBD) binds to ACE2 with 10 to 20 time’s higher affinity. Any mutation in the spike protein gene can lead to enhanced infectivity and severity. Therefore, contemporary research is more focused on identifying new emerging variants and studying their pathogenesis and antigenicity [15–18]. The CoG-UK (Covid-19 Genomics UK) consortium reports that over 4,000 mutations have been identified in the spike glycoprotein to date [19]. Due to these mutations, over 300 naturally occurring variants have been reported from different parts of the globe. However, merely 13 amino acid residues were detected with more than 0.1% mutation rate [20]. Several variants of different lineages with spike glycoprotein mutations have been emerged recently and generated therapeutic concerns, specifically during the ongoing vaccine trialing (COG-UK report, accessed on 15.01.2021). Among these, D614G, A222V of lineage B.1.177, N439K of lineage B.1.258, Y453F, E484K, N501Y of lineage B.1.1.7, N501Y.V2 and lineage P.1 are notable ones.

The B.1 lineage SARS-CoV-2 D614G variant features a non-synonymous mutation, replacing aspartic acid with glycine located at residue 614 of the spike protein, and three point mutations at positions 241, 3,037, and 14,408 [17, 21]. Substituting aspartic acid at position 614 with glycine removes the side-chain hydrogen bond of D614 and T859, resulting in glycosylation of the adjacent N616 site in the S2 domain [17]. This enhances the engagement of the S2 subunit with TMPRSS2, cleavage of the S1 domain, and viral invasion of the host cell [22]. The strain is widespread in Europe, North America, Australia, and Asia, and its infection resulted in rapid transmissibility and increased conglomeration of virus in the upper respiratory tract up to 70%.

Another notable variant is popularly called the UK variant or B.1.1.7. It is a combination of the N501Y mutation (amino acid change from asparagine to tyrosine at position 501 in the S gene) along with a 6-base deletion that removes histidine and valine at positions 69 and 70 (69-70del) [23]. The earliest detection of the mutation occurred in April 2020 in the USA and Brazil. Later, a mutant with both the N501Y RBD mutation and the deletion was identified on 20th September 2020 in the UK, leading to inflated COVID-19 infection numbers and confusion among scientists and politicians. Initially, the variant was classified as a variant under investigation (VUI-202012/01) and later reclassified as a variant of concern (VOC-202012/01) in December 2020. This variant belongs to 20B/GR clade and the B.1.1.7 lineage, has a total of 23 mutations, including 6 synonymous mutations, 13 non-synonymous mutations, and 4 deletions. Mutation N501Y increases receptor binding affinity and enhances viral transmissibility up to 70% in combination with 69-70del [24]. As the mutation is in the RBD, it also influences the efficacy of antibody neutralization.

Since the emergence of the pandemic, several phytocompounds were substantiated due to their antiviral properties for inhibiting SARS-CoV-2 viral entry and replication [11, 25–27]. Computational tools in this regard deserve special attention because of their efficiency in screening accurately the antiviral potential of phytocompounds in silico. It is not only cost-effective but also time-saving in screening the antiviral potential of phytochemicals in comparison to wet laboratory experiments. Our recent computational analyses demonstrated inhibition of the S protein binding to ACE2 because of strong binding affinities of polyphenolic compounds like catechin, curcumin, and 8-Hydroxydihydrosanguinarine (8-HDS) to ACE2, S protein, and RBD/ACE2 complex of SARS-CoV-2 [28, 29]. Catechin and curcumin interact at the junction of the RBD/ACE2 complex, triggering fluctuations in its alpha helices and beta strands. Catechin has more binding affinity than curcumin and binds to the proximity of the RBD, whereas curcumin binds directly with the RBD [29]. Likewise, the derivative of sanguinarine, 8-HDS, also exhibited excellent viral entry inhibitory potential by causing structural perturbations within the secondary structures of the M and S proteins. The pyridone ring in 8-HDS can impede the enzymes essential for viral replication [28]. However, with the rise of multiple new variants exhibiting increased transmissibility, the prospective effectiveness of these compounds in blocking viral entry requires reevaluation.

Further, to avoid the time consumption in designing a new drug after identifying any new compound, present-day research is more inclined towards repurposing drugs with existing compounds and their derivatives. Curcumin, being both lipophilic and hydrophobic, has several constraints related to its bioavailability, and its analogues have been elucidated with better bioactivity potential [30]. Among the two tautomeric forms of curcumin, the keto-form has better antioxidant activity than the enol-form [31]. Thus, in the present study, we have considered a derivative of curcumin, i.e., half-curcumin (half of the keto-form of curcumin), along with our previously examined phytocompounds (catechin, curcumin, and 8-HDS). These compounds and their derivatives are assessed for their efficacy in intervening viral entry of D614G-mutated strain and the B.1.1.7 variant of SARS-CoV-2. The derivative half-curcumin is evaluated for its drug likeness and suitability and compared with curcumin.

## Materials and Methods

### Sequence Analysis

Multiple sequence alignment was carried out using the spike protein FASTA sequences of 2019-nCoV, D614G, and B.1.1.7. The alignment results of 2019-nCoV showed that all three chains of the spike protein share the same amino acid sequence, only a single chain was selected for secondary structure analysis and physicochemical property prediction. Since the D614G and B.1.1.7 strains do not share identical sequences, the physicochemical properties were averaged across all chains.

## Molecular Interaction Study

Molecular docking analyses using AutoDock Tools 1.5.6 were performed to evaluate the binding free energies of the D614G and B.1.1.7 S proteins with curcumin, half-curcumin, catechin, and 8-HDS. PubChem was used to obtain the canonical SMILES IDs of catechin (Catechin–Gallocatechin–Catechin), curcumin, and 8-HDS (https://pubchem.ncbi.nlm.nih.gov/). The structure of the half-curcumin was designed using ChemSketch. Conversions to 3D structures were done using CHIMERA 1.11.2 [32]. Structures of the spike protein from 2019-nCoV (PDB ID: 6VSB) and its D614G mutant (PDB ID: 7KDL), with resolutions of 3.46 Å and 2.96 Å, were collected from the PDB. S-protein three-dimensional structure of B.1.1.7 also retrieved from GISAID (https://www.gisaid.org/, accessed on 20th January, 2021). Water molecules and salt ions were removed from the protein structures using Discovery Studio 2017 R2 Client to ensure a clean and optimized system suitable for computational analysis and interaction modeling. The interactions between the spike protein and ligands—curcumin, 8-HDS, catechin, and half-curcumin—were measured for binding affinity using AutoDock Vina 1.1.2 [33]. The binding site of the spike protein was characterized by evaluating binding affinity, receptor-interacting and pocket atoms, interaction sites, atomic contact energy (ACE), and side-chain residues. The outcomes of the docking analyses were examined and displayed through Discovery Studio 2017 R2 Client [34].

## Protein-Protein Interaction

ClusPro 2.0, a computerized rigid-body docking program, was employed to analyze S-protein–ACE2 interactions in the presence and absence of curcumin, 8-HDS, catechin, and half-curcumin. Docked conformations were analyzed with respect to their clustering properties using this tool, which takes different protein parameters into account. Selection of conformations was guided by empirical free energy calculations, including both desolvation and electrostatic energies. The ClusPro server is available at https://cluspro.bu.edu/publications.php. As an FFT-based rigid docking tool, Piper supports the ClusPro clustering program by generating 1,000 low-energy conformations to identify the native binding site [35]. The native site is considered to span a broad range of free energies to capture a larger set of results. Initially, the ligand was sampled at approximately 10^9^ positions relative to the receptor, from which only the top 10³ positions were selected for further analysis.

## Drug Likeliness Analysis

SwissADME is a comprehensive online platform that provides detailed insights into a compound’s physicochemical characteristics, pharmacokinetic behavior, drug-likeness, and medicinal chemistry properties. By evaluating parameters such as absorption, distribution, metabolism, excretion (ADME), and toxicity, it enables researchers to assess the potential of a molecule as a drug candidate and predict its behavior in biological systems. The Bioavailability Radar, which evaluates key physicochemical parameters such as lipophilicity, molecular size, polarity, solubility, saturation, and flexibility, was employed to assess the drug-likeness of compounds. Furthermore, a comprehensive evaluation of ADME properties—absorption, distribution, metabolism, and excretion—is crucial in drug development, as it helps predict a molecule’s pharmacokinetic behavior, potential efficacy, and safety profile in biological systems. The molecular structure of half-curcumin was constructed using ChemSketch, and its ADME properties were subsequently predicted using SwissADME (http://www.swissadme.ch/).

## Results

### Structural Analysis

The secondary structures of the 2019-nCoV, D614G, and B.1.1.7 spike proteins were predicted using SOPMA (Self Optimized Prediction Method with Alignment), which revealed distinct variation in all the three strains. *α*-Helix contents of S protein in 2019-nCoV, D614G, and B.1.1.7 was 27.17%, 27.47%, and 25.68%, respectively. In our study, we hardly observed any differentiation in positive amino acids among the variants, the % of negatively charged amino acids were observed in the following manner, 2019-nCoV < B.1.1.7 < D614G variant. The half-life of a protein refers to the duration required for it to be fully degraded following its synthesis in a cell. For the 2019-nCoV spike protein, the maximum half-life in mammalian reticulocytes was estimated to be 30 h. But, in in vitro condition, the half-life of chain Aof S protein of D614G strain was 4.4 h and 1.2 h for both chain B and C. Similarly, for B.1.1.7 variant, the half-life of the S protein was computed as 1 h. The instability index of S protein was the highest for 2019-nCoV (31.58), followed by D614G (30.57) and B.1.1.7 (29.205). However, the S protein of the D614G strain (85.46) has a higher aliphatic index as compared to that of 2019-nCoV (81.58) and B.1.1.7 (80.21). The average GRAVY value was − 0.163, − 0.049, and − 0.054 for 2019-nCoV, B.1.1.7, and D614G strains, respectively.

## Molecular Interaction Study

**Fig. 1:**
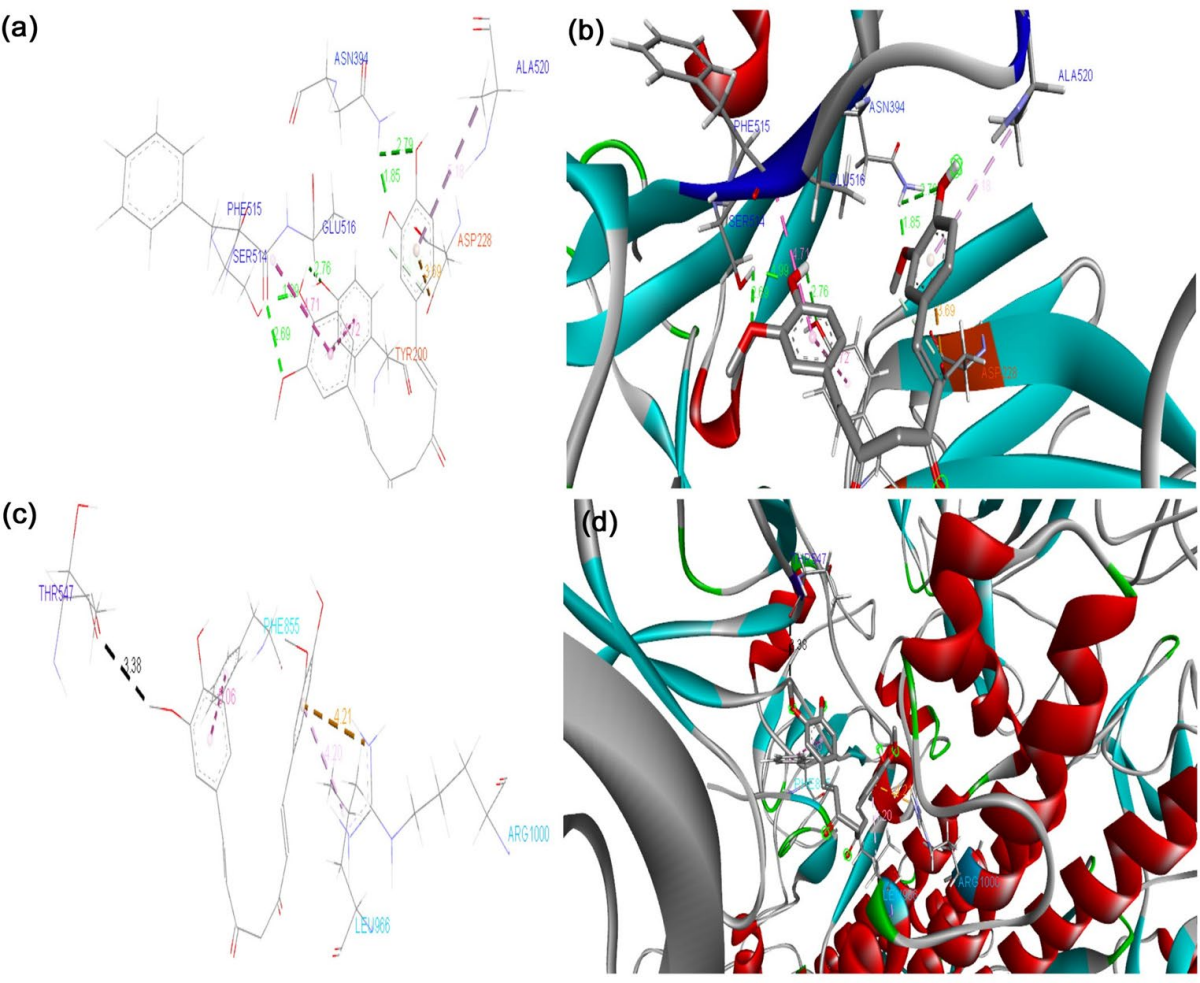
Docked conformation of curcumin within the binding pocket of S Protein variants.** a** Amino acids involved in curcumin interaction with the D614G variant,** b** Types of bonds formed between curcumin and the D614G S Protein within the binding pocket,** c** Amino acids involved in curcumin interaction with the B.1.1.7 variant,** d** Types of bonds formed between curcumin and the B.1.1.7 S Protein within the binding pocket. This figure was produced using Discovery Studio Visualizer (http://accelrys.com/products/collaborative-science/biovia-discovery-studio/visualization-download.php)

**Fig. 2:**
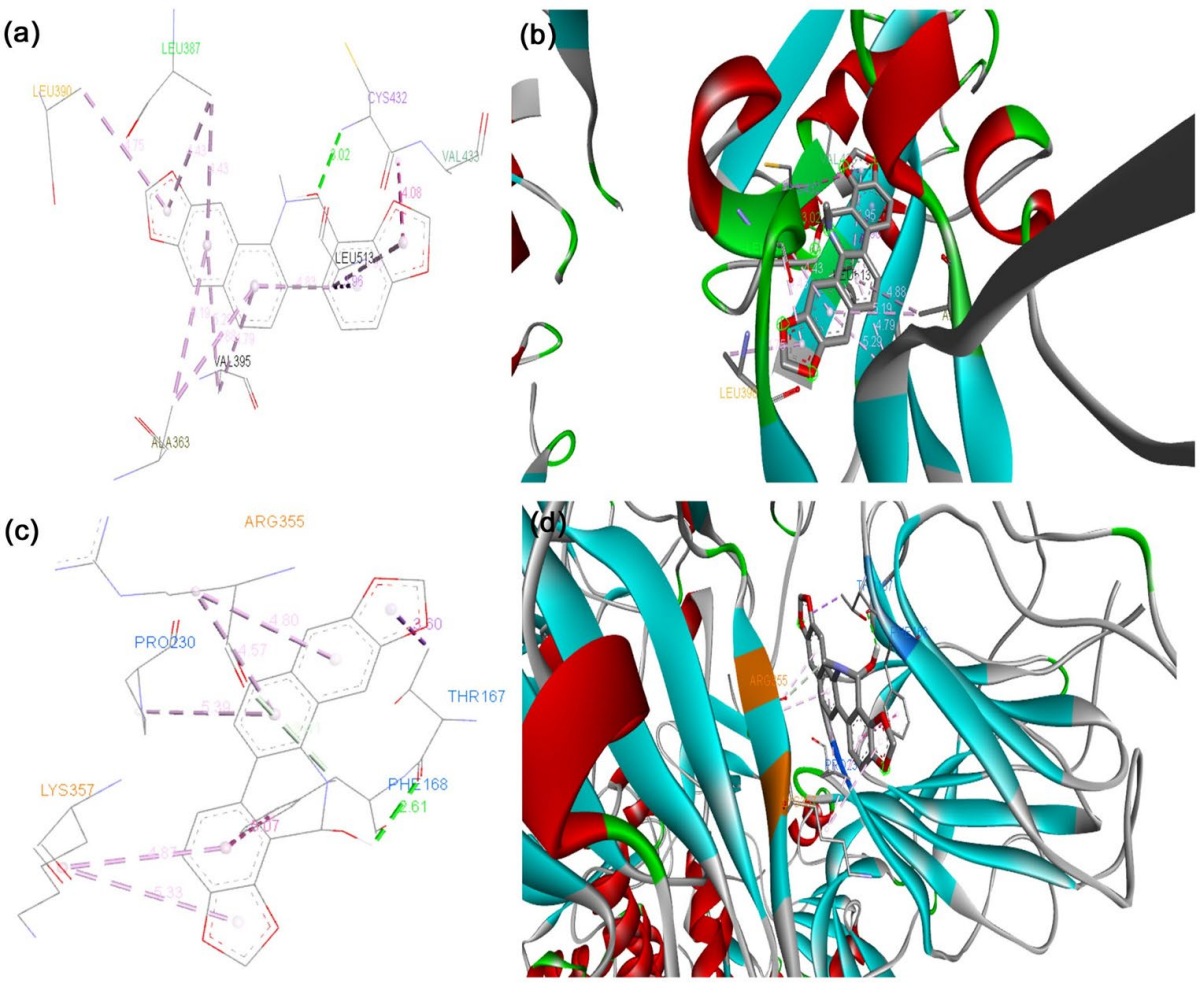
Docked conformation of 8-HDS within the binding pocket of S Protein variants.** a** Amino acids involved in the interaction between 8-HDS and the D614G S Protein,** b** Types of bonds formed between 8-HDS and the D614G S Protein in the binding pocket,** c** Amino acids involved in the interaction between 8-HDS and the B.1.1.7 S Protein,** d** Types of bonds formed between 8-HDS and the B.1.1.7 S Protein in the binding pocket. This figure was produced using Discovery Studio Visualizer (http://accelrys.com/products/collaborative-science/biovia-discovery-studio/visualization-download.php)

**Fig. 3:**
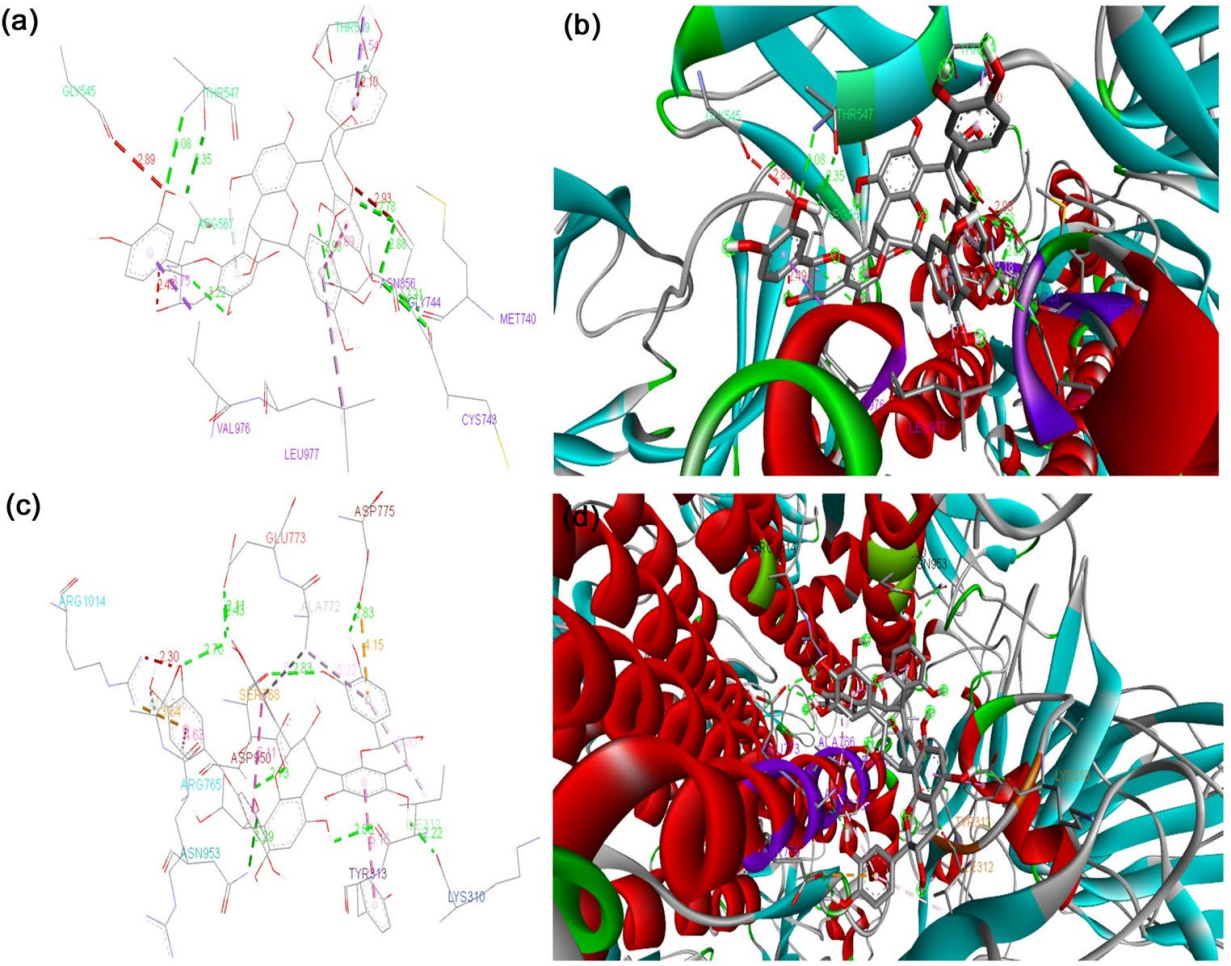
Docked conformation of catechin within the binding pocket of S Protein variants. **a** Amino acids involved in catechin interaction with the D614G S Protein,** b** Types of bonds formed between catechin and the D614G S Protein in the binding pocket,** c** Amino acids involved in catechin interaction with the B.1.1.7 S Protein, **d** Types of bonds formed between catechin and the B.1.1.7 S Protein in the binding pocket. This figure was produced using Discovery Studio Visualizer (http://accelrys.com/products/collaborative-science/biovia-discovery-studio/visualization-download.php)

**Fig. 4:**
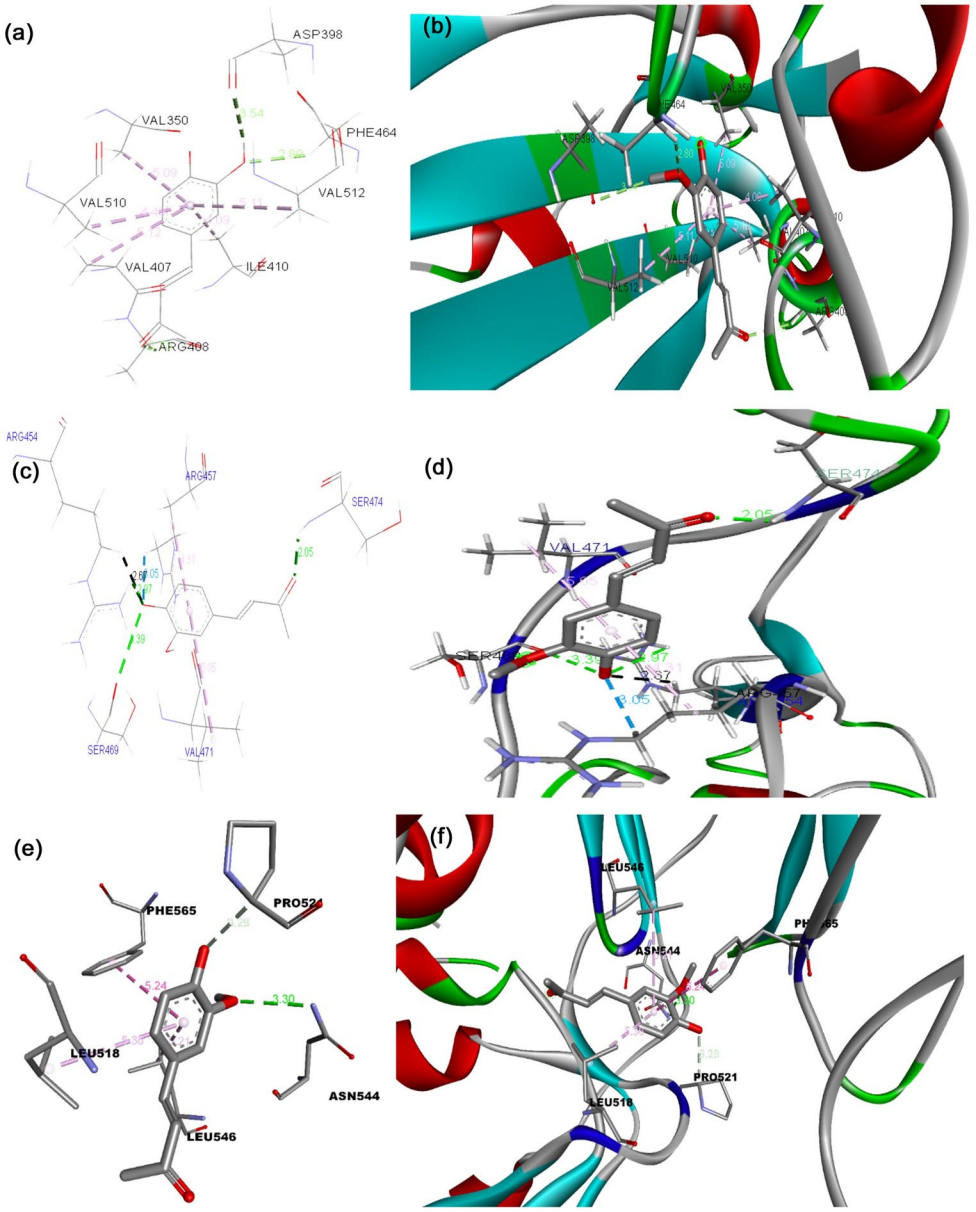
Docked conformation of half-curcumin within the binding pocket of the S Protein of 2019-nCoV and its variants.** a** Amino acids involved in interaction with the D614G S Protein,** b** Types of bonds formed with the D614G S Protein in the binding pocket,** c** Amino acids involved in interaction with the B.1.1.7 S Protein,** d** Types of bonds formed with the B.1.1.7 S Protein in the binding pocket,** e** Amino acids involved in interaction with the 2019-nCoV S Protein,** f** Types of bonds formed with the 2019-nCoV S Protein in the binding pocket. This figure was produced using Discovery Studio Visualizer (http://accelrys.com/products/collaborative-science/biovia-discovery-studio/visualization-download.php)

The binding energy of the S protein of the D614G strain with the phytocompounds and their derivatives, curcumin, 8-HDS, catechin, and half-curcumin, scored − 7.0 Kcal mol^− 1^, -10.5 Kcal mol^− 1^, − 9.9 Kcal mol^− 1^, and − 6.4 Kcal mol^− 1^, respectively. The binding affinity of curcumin, 8-HDS, catechin, and half-curcumin with the S protein of B.1.1.7 was noted as -8.0 Kcal mol^− 1^, − 9.6 Kcal mol^− 1^, − 10.5 Kcal mol^− 1^, and − 6.2 Kcal mol^− 1^, respectively (Table 1). Curcumin interacts via a hydrogen bond with the S protein of D614G at the amino acid residues, ASN856, MET740, GLY744, CYS743, THR547, and ARG567 (Fig. 1a, b), likewise with the S protein of B.1.1.7 at THR547 (Fig. 1c, d). The sanguinarine derivative 8-HDS forms a Hydrogen bond with CYS432 and THR167 S protein amino acid residues of D614G (Fig. 2a, b) and ARG355 amino acid residue of B.1.1.7 (Fig. 2c, d). Catechin binds to the S protein of D614G through Hydrogen bonds with ASN1023 and ARG1039 residues (Fig. 3a, b). It also makes a Hydrogen bond with the RBD fragment of B.1.1.7 at SER768, ASP775, GLU773, LYS310, ILE312, ASN953, ASP950, and ALA766 amino acids (Fig. 3c, d). Similarly, half-curcumin forms a Hydrogen bond with the amino acid residues of the S protein of D614G at ASP398 and SER474 residues (Fig. 4a, b), and ARG454 and SER469 amino acid residues of B.1.1.7 (Fig. 4c, d).

**Table 1:** The binding energies, types of interactions, and the amino acid residues involved in the interaction between the S protein of the D614G variant, B.1.1.7 variant, and the original 2019-nCoV with various phytochemicals and their derivatives

Protein- ligand	Protein- ligand 2D interaction	Binding affinity (Kcal/mol)	Interaction	AA: name; AA: No
D614G – curcumin		**− 7.0**	**Van der Waal**	THR572, THR573, ILE587, PHE541, GLY550, GLY548, ASN540, ASP745, ASN978, SER746, TYR741, ARG1000, ASP979, ILE742
			**Conventional hydrogen bond**	ASN856, MET740, GLY744, CYS743, THR547, ARG567
			**Unfavourable donor donor**	THR549, GLY545
			**Pi- Sigma**	VAL976
			**Pi-Alkyl**	LEU546, LEU977
D614G – 8-HDS		− 10.5	**Van der Waal**	SER514, PRO384, PAE515, GLY431, TYR365, VAL433, PHE392, CYS391, VAL524, PHE338, PHE342, VAL511
			**Conventional Hydrogen Bond**	CYS432
			**Pi- Sigma**	LEU513
			**Pi-Alkyl**	ILE434, VAL395, ALA363, LEU387, LEU390
D614G – catechin		− 9.9	**Van der Waal**	SER1021, ALA1020, ALA1020, THR1027, ASN1023, ARG1039, THR1027, PHE1042, ARG1039, THR1027
			**Conventional Hydrogen Bond**	ASN1023, ARG1039
			**Pi- Sigma**	LEU1024, LEU1024
D614G – half-curcumin		− 6.4	Van der Waal	ALA435, ALA411, ARG408, ILE434, PRO412, VAL433, VAL511, PHE464, TYR423, TRP353, PHE400
			Carbon Hydrogen Bond	ASP398
			Pi-Alkyl	VAL512, VAL510, VAL350, VAL407, ILE410
B.1.1.7 – curcumin		− 8.0	**Van der Waal**	TYR741, MET740, ILE742, GLY744, CYS743, LEU977, PHE572, VAL976, SER975, SER967, ASP571, THR573, LEU546, GLY548
			**Carbon Hydrogen Bond**	THR547
			**Pi-Cation**	ARG1000
			**Pi-Sigma**	LEU966
			**Pi-Pi T-Shaped**	PHE855
B.1.1.7 – catechin		− 10.5	**Van der Waal**	LYS733, ARG776, ILE770, ALA771, GLY769, GLN954, LEU1012, PRO665, ILE664, GLY311, GLN314, VAL303, GLN949, GLN957
			**Conventional Hydrogen Bond**	SER768, ASP775, GLU773, LYS310, ILE312, ASN953, ASP950
			**Carbon Hydrogen Bond**	ALA766
			**Unfavourable Donor Donor**	ARG1014
			**Pi-Alkyl**	ALA772
			**Pi-Pi T-Shaped**	TYR313
			**Amide-Pi Stacked**	ARG765
B.1.1.7 – half-curcumin		− 6.2	**Van der Waal**	GLY477, PHE473, PRO472, HIS458, LEU456, ASP467
			**Conventional Hydrogen Bond**	SER474, ARG454, SER469
			**Pi-Alkyl**	ARG457, VAL471
B.1.1.7–8-HDS		− 9.6	**Van der Waal**	ILE468, GLU169, TRP353, ARG466, LEU231
			**Conventional Hydrogen Bond**	THR167
			**Carbon Hydrogen Bond**	ARG355
			**Pi-Pi T-Shaped**	PHE168
			**Pi-Alkyl**	PRO230, LYS357
2019-nCoV – half-curcumin		− 6.2	**Van der Waal**	LEU390, CYS391, LEU517, GLY545, ALA520, GLN564, ALA522, VAL576, PHE543, ASP979
			**Conventional Hydrogen Bond**	ASN544
			**Carbon Hydrogen Bond**	PRO521
			**Pi-Pi T-Shaped**	PHE565
			**Pi-Alkyl**	LEU546, LEU518

## Protein-Protein Interaction

**Fig. 5:**
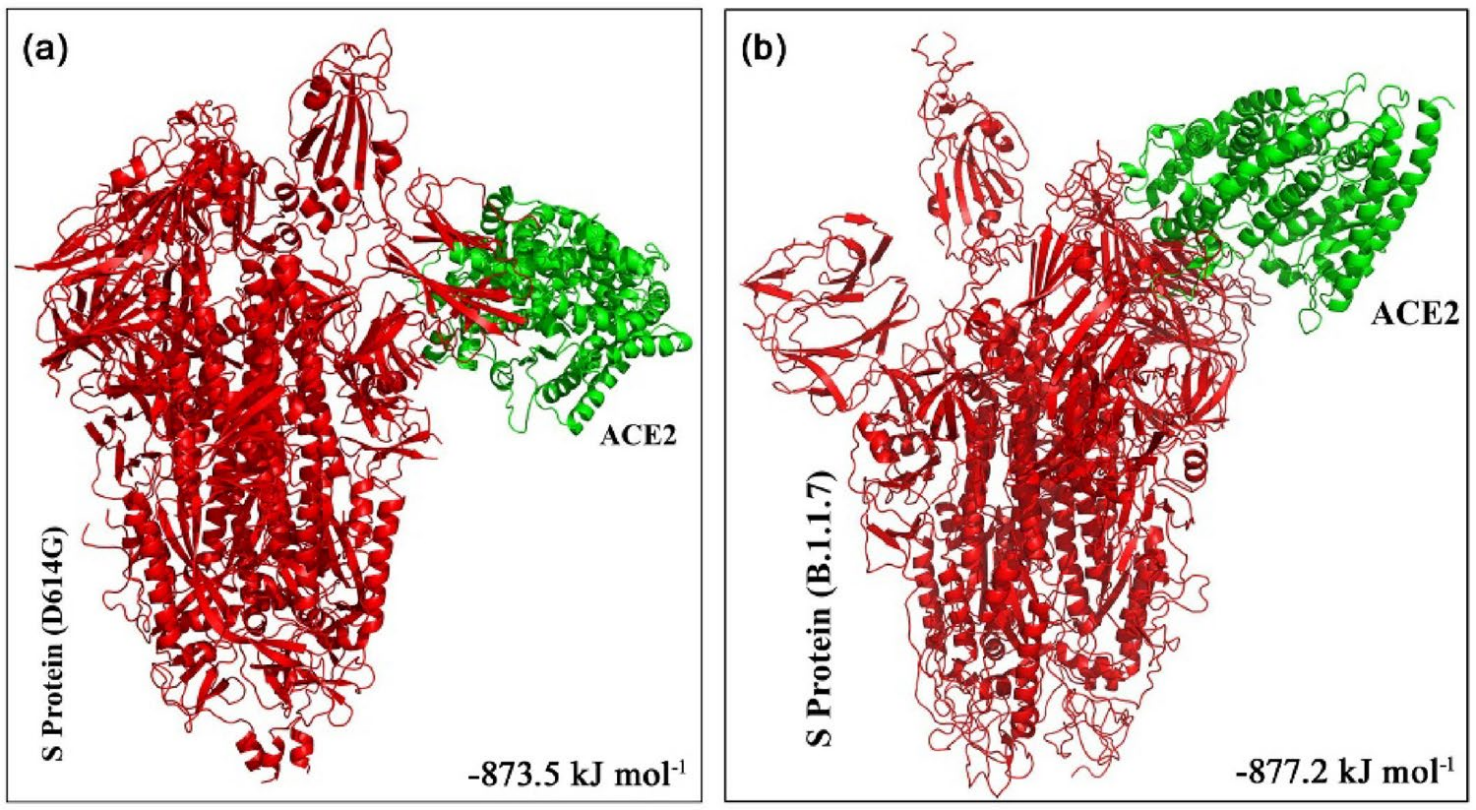
Docked model depicting interaction of S Protein of **a** D614G variant and** b** B.1.1.7 variant with ACE2 receptor in the absence of polyphenols.

**Fig. 6:**
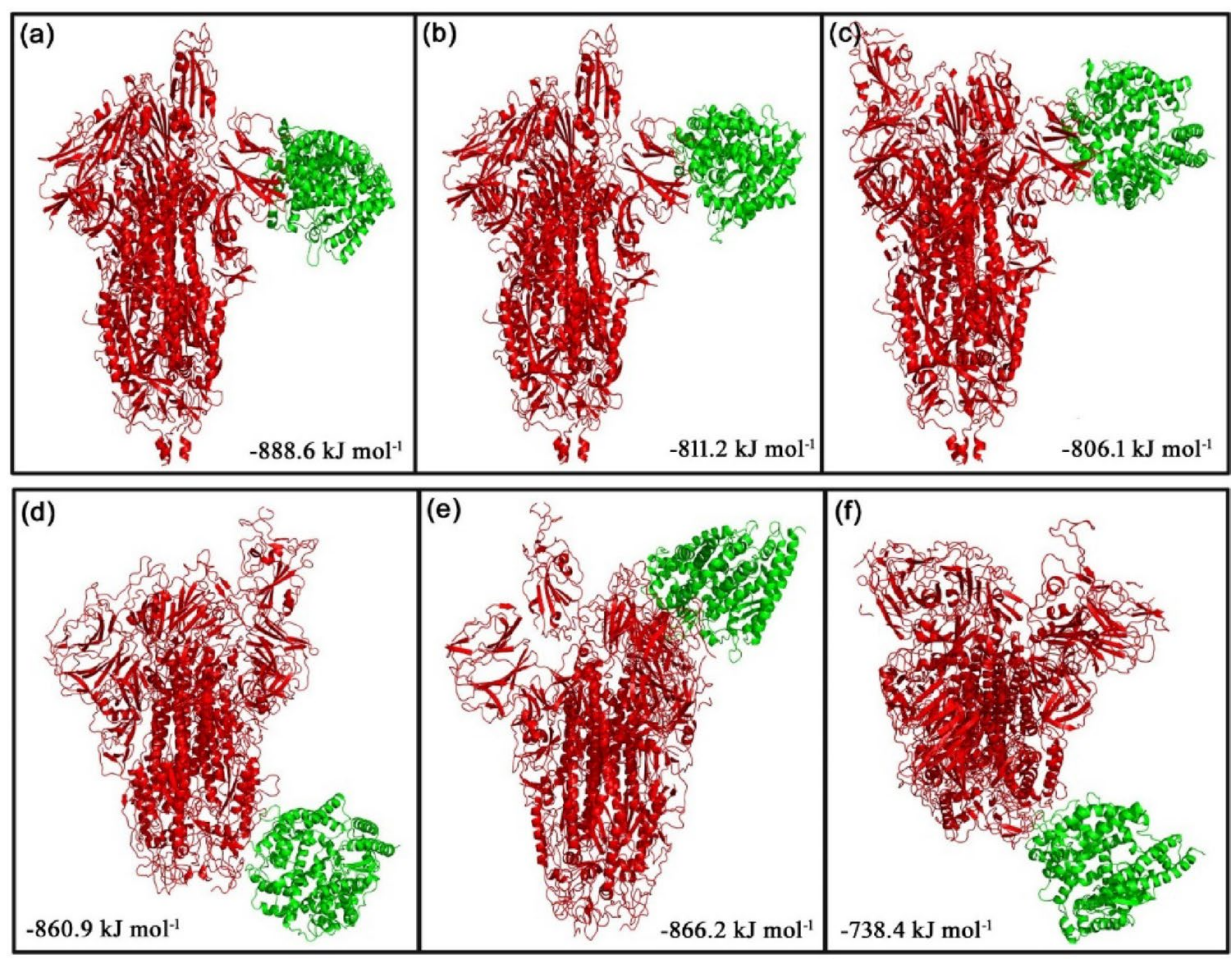
The top 3 docked models displaying interaction of** a**–**c** S Protein of D614G and** d**–**f** S Protein of B.1.1.7 with ACE2 receptor in the presence of curcumin

**Fig. 7:**
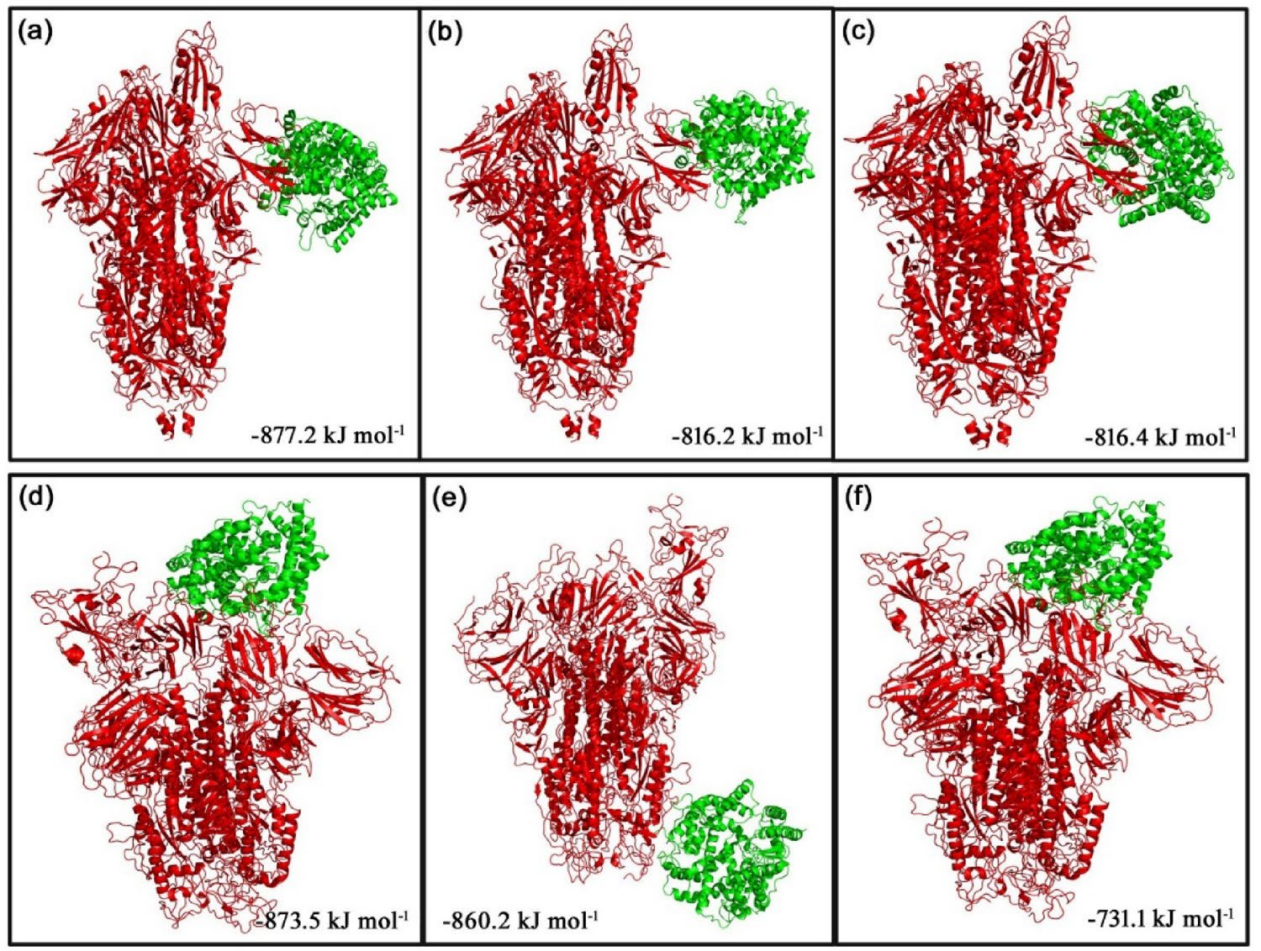
The top 3 docked models displaying interaction of** a**–**c** S Protein of D614G and** d**–**f** S Protein of B.1.1.7 with ACE2 receptor in the presence of catechin

**Fig. 8:**
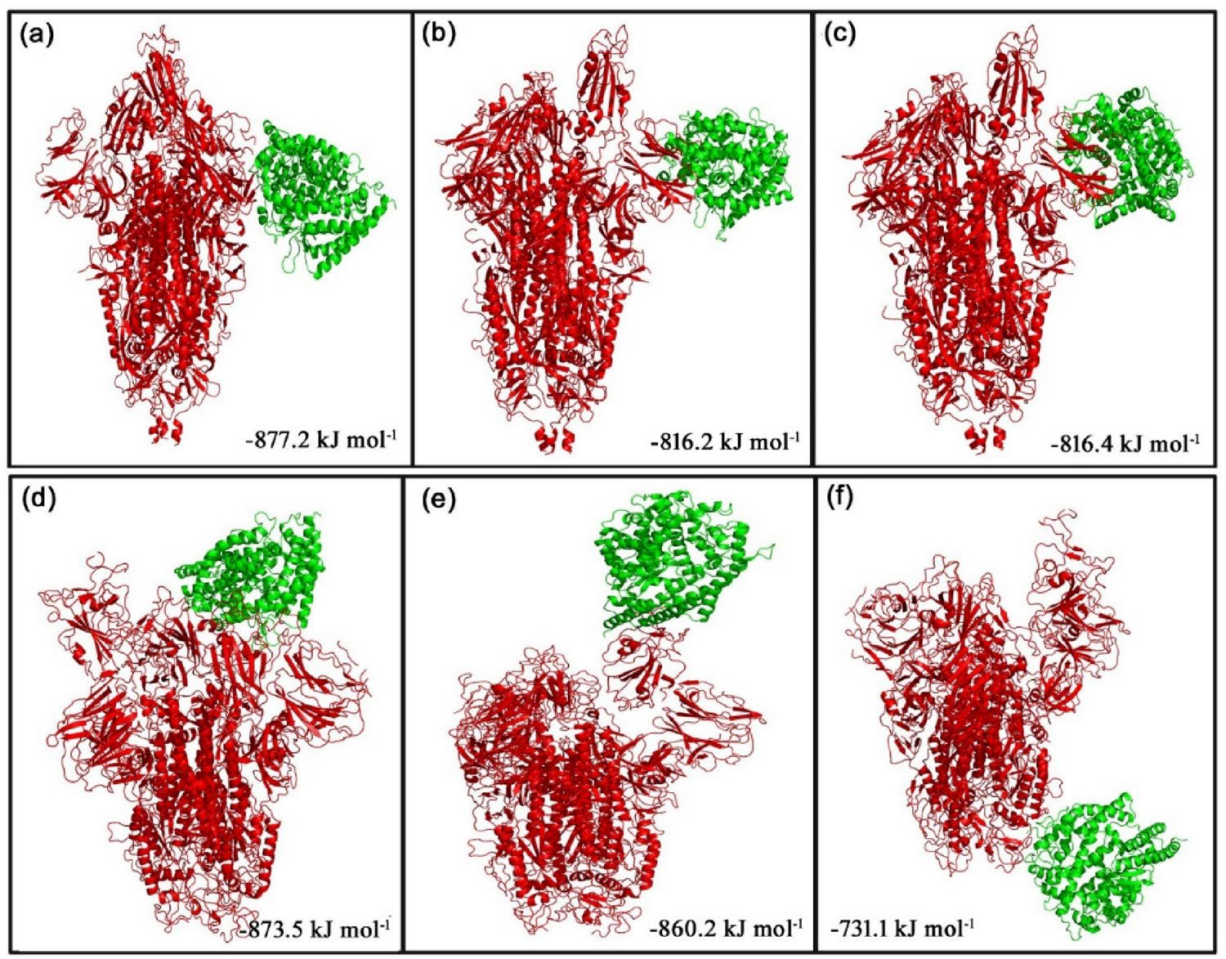
The top 3 docked models displaying interaction of ** a**–**c** S Protein of D614G and ** d**–**f** S Protein of B.1.1.7 with ACE2 receptor in the presence of 8-HDS

**Fig. 9:**
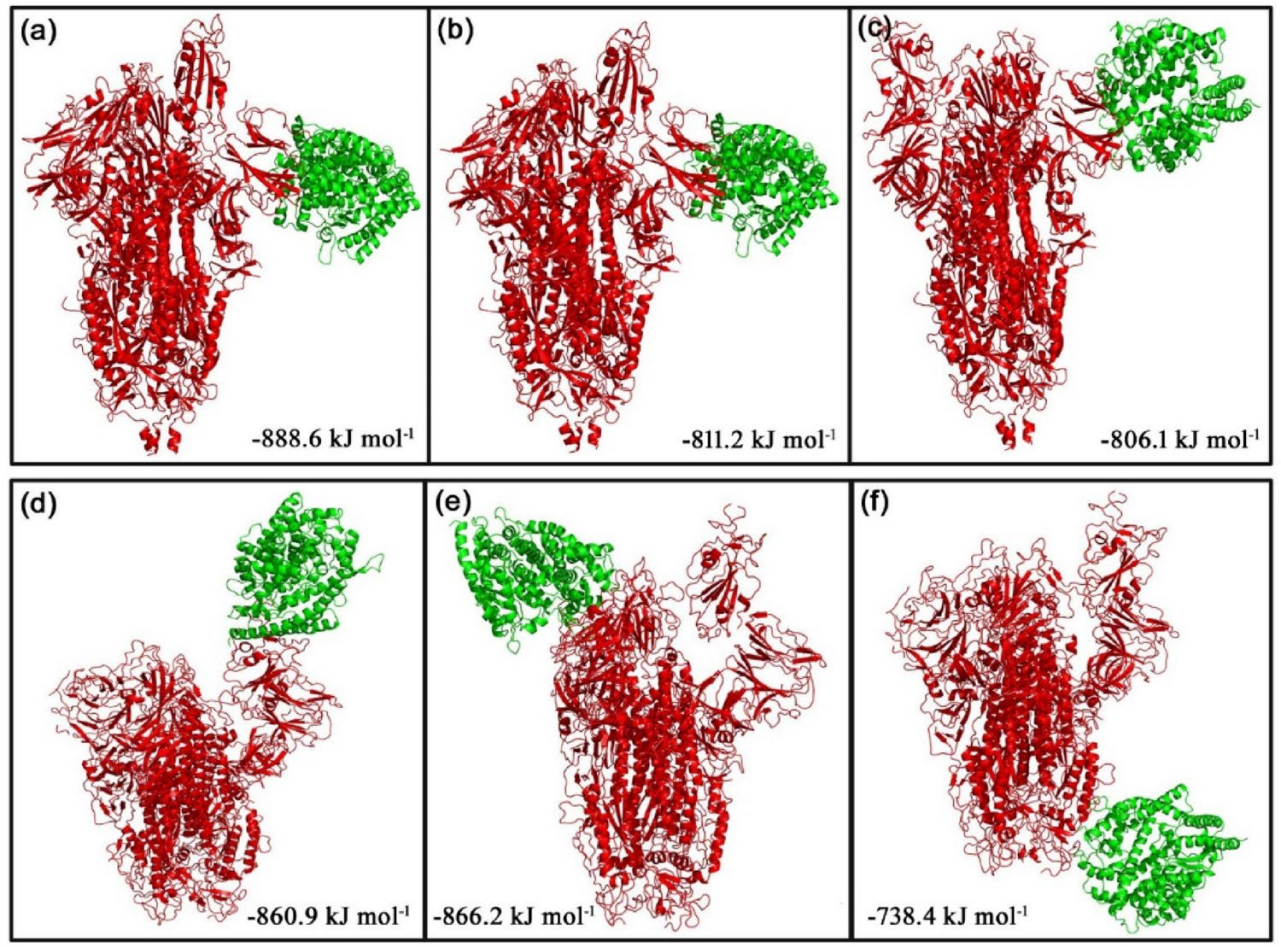
The top 3 docked models displaying interaction of ** a**–**c** S Protein of D614G and ** d**–**f** S Protein of B.1.1.7 with ACE2 receptor in the presence of half-curcumin

The ClusPro web server was used to generate the top 10 docking models exhibiting the most favorable free energy scores. For clustering these models, the total root-mean-square deviation (RMSD) was employed as a key criterion, allowing the identification of structurally similar conformations and facilitating the selection of representative docked poses [36]. In this study, three ClusPro docking models were analyzed, selected according to the likelihood of interactions between the S protein of each strain and the phytocompounds or their derivatives, as well as their binding to the predicted ACE2 interaction sites. The mean binding energy across the three binding positions for the D614G S protein–ACE2 interaction is − 846.6 kJ mol^− 1^. However, when 8-HDS or catechin is present individually, the average binding energy of the S protein–ACE2 complex is − 836.6 kJ mol^− 1,^ and in the presence of curcumin and half-curcumin, individually, is − 835.3 kJ mol^− 1^. Similarly, the S protein of B.1.1.7 interacts with ACE2 with a binding energy of − 831.6 kJ mol^− 1^. In contrast, the average binding energy of the S protein–ACE2 complex in the simultaneous presence of 8-HDS and catechin is -821.6 kJ mol^− 1^ and in the presence of curcumin and half-curcumin is − 821.8 kJ mol^− 1^ (Table 2; Figs. 5, 6, 7, 8 and 9).

**Table 2:** Protein–protein interactions showing the three lowest binding energies and the average minimum energy of the S Protein–ACE2 complex, both in the presence and absence of phytochemicals and their derivatives

Macromolecule	Binding positions	Lowest energy (kJ/mol)	Average lowest energy (kJ/mol)
D614G-ACE2	1	− 877.2	− 846.6
D614G-ACE2	2	− 822.2	
D614G-ACE2	3	− 820.4	
D614G with 8HDS-ACE2	1	− 877.2	− 836.6
D614G with 8HDS-ACE2	2	− 816.2	
D614G with 8HDS-ACE2	3	− 816.4	
D614G with catechin-ACE2	1	− 877.2	− 836.6
D614G with catechin-ACE2	2	− 816.2	
D614G with catechin-ACE2	3	− 816.4	
D614G with curcumin-ACE2	1	− 888.6	− 835.3
D614G with curcumin-ACE2	2	− 811.2	
D614G with curcumin-ACE2	3	− 806.1	
D614G with half-curcumin-ACE2	1	− 888.6	− 835.3
D614G with half-curcumin-ACE2	2	− 811.2	
D614G with half-curcumin-ACE2	3	-806.1	
B.1.1.7 with 8HDS-ACE2	1	− 873.5	− 821.6
B.1.1.7 with 8HDS-ACE2	2	− 860.2	
B.1.1.7 with 8HDS-ACE2	3	− 731.1	
B.1.1.7 with catechin-ACE2	1	− 873.5	− 821.6
B.1.1.7 with catechin-ACE2	2	− 860.2	
B.1.1.7 with catechin-ACE2	3	− 731.1	
B.1.1.7 with curcumin-ACE2	1	− 860.9	− 821.8
B.1.1.7 with curcumin-ACE2	2	− 866.2	
B.1.1.7 with curcumin-ACE2	3	− 738.4	
B.1.1.7 with half-curcumin-ACE2	1	− 860.9	− 821.8
B.1.1.7 with half-curcumin-ACE2	2	− 866.2	
B.1.1.7 with half-curcumin-ACE2	3	− 738.4	
B.1.1.7-ACE2	1	− 873.5	− 831.6
B.1.1.7-ACE2	2	− 865.2	
B.1.1.7-ACE2	3	− 736.1	

### Drug Likeness

**Fig. 10:**
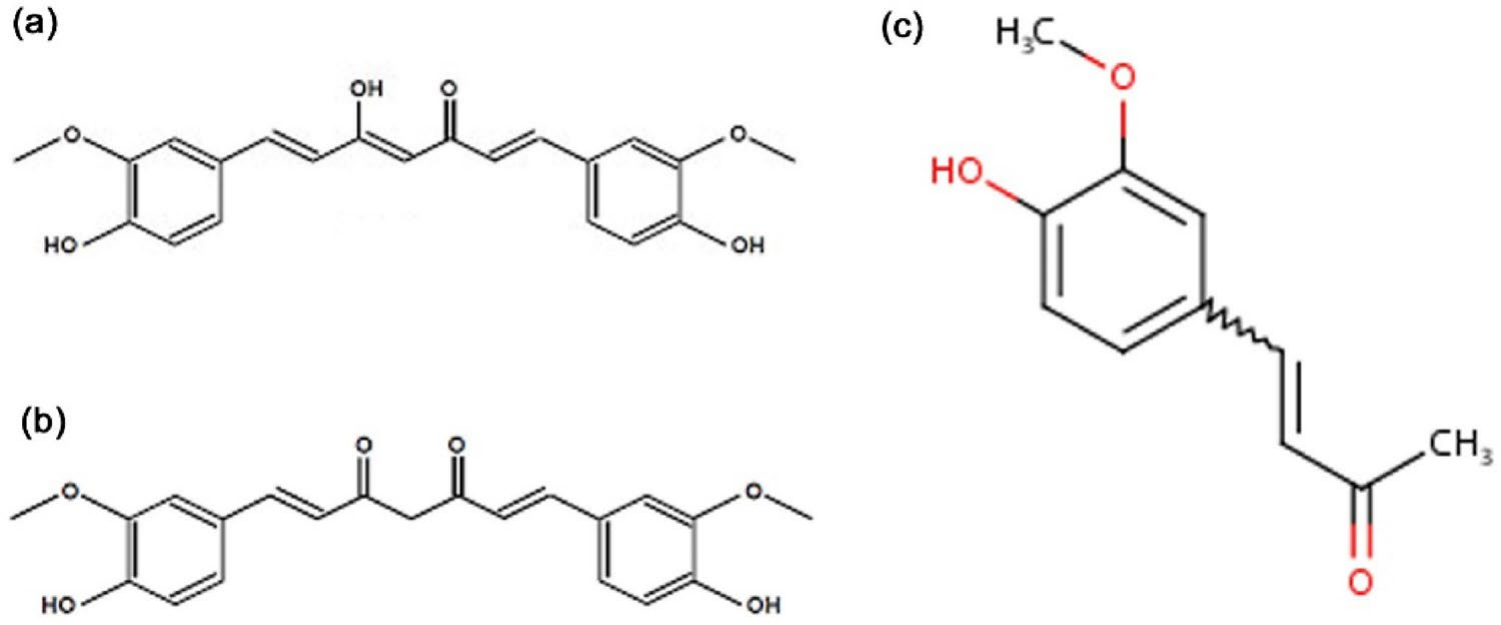
Structure of curcumin and half-curcumin,** a** enol-form of curcumin,** b** keto-form of curcumin and** c** half-curcumin

The SwissADME web tool was employed to evaluate the drug-likeness properties of curcumin and its derivative, half-curcumin. This platform provides a comprehensive assessment of a compound’s pharmacokinetic parameters, physicochemical characteristics, and medicinal chemistry friendliness, enabling the prediction of its suitability as a potential drug candidate. (Fig. 10). This tool analyzes the interplay between the pharmacokinetic and physicochemical properties of the lead compound. Physicochemical properties of curcumin (C_21_H_20_O_6_) and half-curcumin (C_11_H_12_O_3_) were determined as 368.38 and 192.21 g mol^− 1^ molecular weight, respectively. The synthesized derivative of curcumin, i.e., half-curcumin has 14 heavy atoms, 3 hydrogen bond acceptors, 1 hydrogen bond donor, and 3 rotatable bonds. The compound is with molar refractivity of 54.86, and topological polar surface area of 46.53 Å^2^. The computed average lipophilicity scores of half-curcumin and curcumin, based on iLOGP, XLOGP3, WLOGP, MLOGP, and SILICOS-IT methods, were 1.85 and 3.03, respectively. The bioavailability score of both molecules is 0.55. However, half-curcumin has improved water solubility compared to curcumin, which increases its efficacy for use as a drug molecule. Among the five models used for predicting drug-likeness, four—Lipinski, Ghose, Veber, and Egan—indicated that half-curcumin has strong potential as a future drug candidate.

## Discussion

The prolific adaptability of coronaviruses for infecting a wide range of hosts and tissue tropism in varied environmental conditions is facilitated through its capability for recombination and mutation [37]. Similar consequences are also engendered in the current pandemic by the periodic encounter of several mutants or variants. To mitigate the stress on the public health sector, the quest for different potential viral inhibitory phytocompounds is ongoing. While studying the structures and physicochemical properties from the spike protein of the novel coronavirus SARS-CoV-2 and its two variants, D614G and B.1.1.7, we highlighted the stability of these proteins. The little inflated *α*-Helix content in D614G strain suggested its superior stability over other two strains. We hypothesize that negatively charged amino acids may play a key role in enhancing protein thermostability by forming intramolecular salt bridges that stabilize the overall structure and reduce the likelihood of denaturation at elevated temperatures [38]. Based on this premise, we further hypothesize that the D614G and compared to other strains, potentially due to alterations in electrostatic interactions that strengthen the spike protein’s structural integrity.

However, both the variants have superior thermostability over the 2019-nCoV. It was also observed that + ve amino acids have not changed significantly in the due course of mutations and variations. An instability index of less than 40 indicates that the protein is stable [38]. It is observed that the stability of the S protein of 2019-nCoV is reducing gradually with the emergence of each variant. The aliphatic index of a protein represents the proportion of its structure occupied by aliphatic side chains, such as alanine, valine, isoleucine, and leucine. Thus, higher aliphatic index values represent the protein’s ability to withstand high temperatures. Cytotoxins (with aliphatic indices ranging from 66.5 to 84.33) contain a high number of hydrophobic amino acids, and their co-presence with polar (charged) residues generates an amphipathic nature of these proteins. This facilitates biological membrane perturbation. The aliphatic indices of the S protein in all strains are significantly high, particularly in the case of D614G, which is a possible rationale behind its high transmissibility. It is noteworthy to mention that the recently isolated UK variant, B.1.1.7, has a lower index value than the parental strain. Due to the weaker thermostability of this new variant, it has a meager possibility of high transmission in warmer environments. However, the lower GRAVY value of the spike protein of B.1.1.7 proved to be more hydrophilic and less polarized in nature than those of the other two strains, which could have resulted from the mutations.

The physicochemical properties of the spike protein, including α-helix content, GRAVY scores, and electrostatic surface features, further influence these interactions. For instance, higher α-helix content may confer structural rigidity, while alterations in hydrophobicity and charged residues can impact surface complementarity and interaction strength with ACE2. Additionally, increased thermostability observed in the D614G variant may allow the spike protein to maintain its functional conformation under physiological or environmental stress, potentially facilitating a higher proportion of open RBD states available for receptor engagement. Collectively, these structural and physicochemical features not only provide mechanistic insight into variant-specific differences in ACE2 binding but also offer valuable information for the design of inhibitors targeting critical spike residues or stabilizing specific conformations, thereby complementing the observed docking trends.

The significant docking scores revealed strong binding affinity of all the studied phytocompounds towards the S protein of both the variants. Curcumin and catechin, which were evinced as potential inhibitors for 2019-nCoV [29], also showed equally high binding affinity towards the S protein of B.1.1.7 and D614G variants. The pyridine-containing 8-HDS showed a better affinity towards the variants than the parental virus. The binding potential of half-curcumin with all the strains (-6.2 for 2019-nCoV and B.1.1.7 variant; -6.4 for D614G variant) can be considered as significant, keeping in mind the small size of the compound. It was observed that 8-HDS and catechin showed stronger binding affinity than curcumin and half-curcumin due to the presence of more functional groups. But, uniqueness was observed in the preference of the binding sites in the S protein of the variants by the newly studied compounds, i.e., 8-HDS and half-curcumin. Both these compounds show direct interaction with RBD fragments, 8-HDS to the D614G strain, and half-curcumin to both the variants. Furthermore, the findings of the present investigation also suggest that the amino acid residues of the S protein that interact with the phytocompounds differ.

Curcumin binds through Van der Waal interactions with amino acid residues of the RBD site of D614G S Protein, i.e., THR572, THR573, ILE587, PHE541, GLY550, GLY548, and ASN540; through conventional hydrogen bonds with THR547 and ARG567; through unfavourable Donor-Donor bonds with THR549 and GLY545; and through Pi-Alkyl bond with LEU546 (Fig. 1a, b). In case of B.1.1.7, binding at the RBD site was observed through Van der Waal interactions with PHE572, ASP571, LEU546, and GLY548; carbon hydrogen bond with THR547 (Fig. 1c, d). The above docking data disclosed that curcumin possesses strong binding with the RBD site of D614G than B.1.1.7. On the contrary, the catechin have less binding affinity towards the RBD site, as it was unable to bind to the RBD region of S protein in D614G strain and binds partially to this region in the S protein of B.1.1.7 with amino acid residues through Van der Waal interactions with GLY311, GLN314, and VAL303; conventional hydrogen bonds with LYS310 and ILE312; pi-pi T-Shaped bond with TYR313 (Fig. 3c, d; Table 1). It shows improved binding of both these compounds with the B.1.1.7 strain than our previous findings [29], where the binding was not to the RBD site, but in its proximity. 8HDS also binds directly with the RBD site by Van der Waal interactions with D614G SER514, PRO384, PAE515, GLY431, TYR365, VAL433, PHE392, CYS391, VAL524, PHE338, PHE342, and VAL511; by conventional hydrogen bond with CYS432; pi-sigma bond with LEU513; pi-alkyl bond with ILE434, VAL395, ALA363, LEU387, LEU390 (Fig. 2; Table 1). Similarly, half-curcumin binds by Van der Waal interactions with amino acid residues of D614G, ALA435, ALA411, ARG408, ILE434, PRO412, VAL433, VAL511, PHE464, TYR423, TRP353, and PHE400; carbon hydrogen bond with ASP398; pi-alkyl bond with VAL512, VAL510, VAL350, VAL407, ILE410 of S protein (Fig. 4a-b; Table 1) and with B.1.1.7 S Protein by Van der Waal interactions with GLY477, PHE473, PRO472, HIS458, LEU456, and ASP467; conventional hydrogen bonds with SER474, ARG454, SER469, Pi-Alkyl ARG457, and VAL471 (Fig. 4c, d; Table 1). Half-curcumin can bind directly with the RBD site S Protein in both the strains. It also interacts with 2019-nCoV with amino acid residues by Van der Waal interactions with LEU390, CYS391, LEU517, GLY545, ALA520, GLN564, ALA522, VAL576, and PHE543; conventional hydrogen bond with ASN544; carbon hydrogen bond with PRO521; pi-pi T-Shaped bond with PHE565; and pi-alkyl bond with LEU546 and LEU518 (Fig. 4e, f). The binding potential of half-curcumin is though supported by less binding energy, yet its preference for the RBD site is noteworthy, which is crucial in host cell binding [39]. Similarly, this molecule binds to specific sites on ACE2 that play a critical role in facilitating viral entry into host cells. By interacting with these key residues, the molecule may potentially interfere with the virus–receptor interaction, thereby hindering the initial stages of infection [40].

The findings of the present computational investigation propose that these natural polyphenols and derivatives may be used to prevent viral infection. It is needless to mention the importance of catechin and curcumin as well acclaimed immuno-stimulants and inducers of autophagy in the removal and neutralization of viral infection leading to viral clearance [41, 42]. In contemporary studies, it is also suggested that the development of pyridine-containing inhibitors could lead to the formulation of anti-coronaviral drugs, as the pyridine-containing ligand improves the compound’s half-life in plasma [43]. This demonstrates the viability of the pyridine-containing sanguinarine derivative, 8-HDS, as a potential inhibitor of viral entry and replication of SARS-CoV-2 and its variants.

Hydrogen (H)-bonds are pivotal in protein folding, protein-ligand interactions, and catalysis [44, 45]. These bonds depict the stability of complexes, crystal engineering, and the overall structure of biomolecules [44]. In this study, we observed that hydrogen bonding plays a crucial role in stabilizing the interactions between the spike protein and phytochemicals, particularly in the D614G and B.1.1.7 variants. These hydrogen bonds contribute significantly to the binding specificity and strength, suggesting their importance in modulating the potential inhibitory effects of these compounds on viral entry. Curcumin, 8-HDS, catechin, and half-curcumin form Van der Waals interactions with the S protein of all the strains. Together with hydrogen bonds, Van der Wall interactions provide a strong bonding, which is lacking in the interaction between the RBD site amino acid residues with catechin. The rest of the compounds showed promising bindings at the RBD site.

The study also inferred that all the ligand molecules reduce the binding affinity between S protein of both D614G and B.1.1.7 strains with ACE2. Curcumin and its derivative, half-curcumin, exhibited tremendous potential for lowering binding affinity compared to 8-HDS and catechin in the S protein and ACE2 interaction for the D614G strain. In B.1.1.7, all ligand molecules reduce the binding affinity between the S protein and ACE2. The reduction in binding affinity between the proteins by 8-HDS and catechin between S protein and ACE2 is -10 kJ mol^− 1^ for D614G strain and − 10.1 in kJ mol^− 1^ for B.1.1.7 strain. Likewise, curcumin and half-curcumin also reduce the binding affinity between S protein-ACE2, i.e., − 11.3 kJ mol^− 1^ for the D614G strain and − 9.9 kJ mol^− 1^ for the B.1.1.7 strain (Table 2). It can be clearly perceived that the significant decrease in the binding energy in the presence of curcumin, catechin, half-curcumin, and 8-HDS suggests their capability in hindering the attachment of the RBD site of the S protein to the ACE2 receptor protein. Thus, the study’s findings would pave the way for further employment of these phytocompounds in the design of effective therapies to prevent viral entry.

**Fig. 11:**
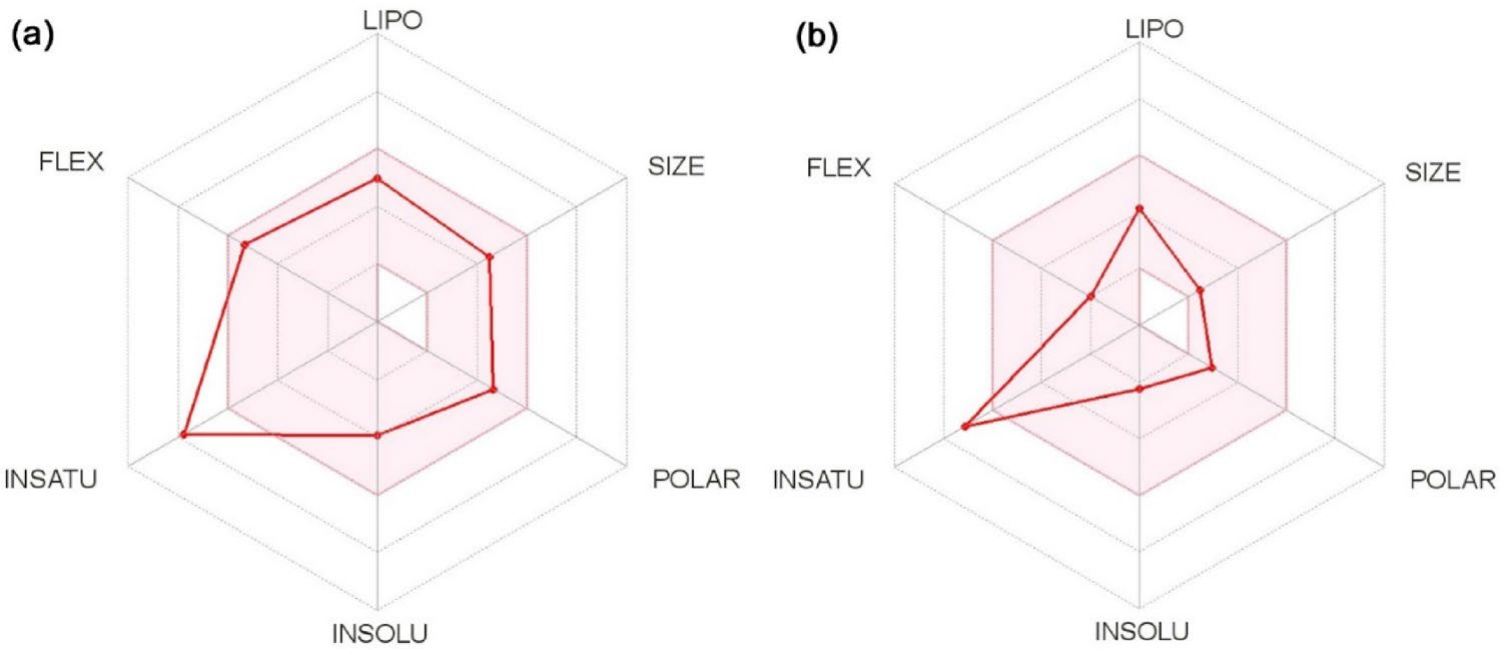
The Bioavailability Radars depicts glimpse of the drug-likeness of** a** curcumin and** b** half-curcumin. The pink area represents the optimal range for each property of both curcumin and its derivative half-curcumin

The two essential aspects of an ideal drug molecule are efficacy, which depict the reach of the molecule in optimized concentration at the target, and bioavailability, which enables its bioactive form till the necessary biological events. The Swiss ADME technology makes the process of drug discovery with more efficient consuming fewer resources. The likelihood of a drug candidate with significant bioavailability can fairly be confirmed on the structural or physicochemical properties of a compound [46]. Optimal range of lipophilicity, size, polarity, solubility, saturation, and flexibility for half-curcumin (Fig. 11) is represented by the bioavailability Radar. The three rotatable bonds in half-curcumin suggest its superior flexibility over curcumin. The BOILED-Egg model [47] also predicts easy penetration of half-curcumin through the blood-brain barrier (BBB) and human gastrointestinal absorption (HIA), showing its promising aptitude towards drug formulation.

Amidst the present health care emergency, the insurgence of several variants/mutants has created unexpected havoc among the public health researchers. The present exploration suggests two reputed phytocompounds, catechin and curcumin, and two analogues, half-curcumin and 8-HDS, as potential viral inhibitors against not only 2019-nCoV, but also against their recent variants, D614G and B.1.1.7. Concurrently, half-curcumin has also exhibited promising drug-like properties, which can mitigate therapeutic needs in this crisis.

## Conclusion

The novel coronavirus has profoundly impacted social, health, and economic domains, creating widespread disruptions in daily life, public health systems, and global economic activities. By infecting millions of people around the world, taking several thousand lives, and shaking socio-economic stability, the COVID-19 pandemic has become a worldwide dismay. This includes an impulse to avoid the march of the increasingly declining scourge. Our research through computational methods shows that 8-HDS and half-curcumin can be an effective inhibitor of various strains of viral infection blockage. It can thus prove to be a potential molecule in the production of therapeutic drugs against COVID-19.

## Data Availability

No datasets were generated or analysed during the current study.
